# Essential oils of *Zingiber officinale*: Chemical composition, *in vivo* alleviation effects on TPA induced ear swelling in mice and *in vitro* bioactivities

**DOI:** 10.3389/fnut.2022.1043175

**Published:** 2022-10-24

**Authors:** Silu Zhang, Lijun Zhang, Mengjie Yu, Donghui Luo, Shuai Chen, Weifeng Liu, Yehui Zhang, Lanyue Zhang, Tiantian Zhao

**Affiliations:** ^1^Key Laboratory of Functional Foods, Ministry of Agriculture and Rural Affairs, Guangdong Key Laboratory of Agricultural Products Processing, Sericulture and Agri-Food Research Institute, Guangdong Academy of Agricultural Sciences, Guangzhou, China; ^2^Shenzhen Precision Health Food Technology Co., Ltd., Shenzhen, China; ^3^School of Traditional Chinese Medicine, Guangdong Pharmaceutical University, Guangzhou, China; ^4^Guangdong Provincial Key Laboratory of Plant Resources Biorefinery, School of Biomedical and Pharmaceutical Sciences, Guangdong University of Technology, Guangzhou, China; ^5^Food Science and Engineering Department, Chaozhou Branch of Chemistry and Chemical Engineering Guangdong Laboratory, Chaozhou, China

**Keywords:** *Zingiber officinale*, edible spices, essential oil, anti-inflammation, anti-bacterial effects, antioxidation, anti-tumor property

## Abstract

*Zingiber officinale* (ZO) is a traditional food condiment. The essential oils of *Z. officinale* (ZOEOs) are known to have multiple bioactivities. In this study, gas chromatography mass spectrometer (GC-MS) analytical method was used to identify active ingredient present in ZOEOs. A total of 41 compounds were identified in ZOEOs. Major components in ZOEOs were zingiberene (19.71%), (+)-β-cedrene (12.85%), farnesene (12.17%), α-curcumene (10.18%) and β-elemene (3.54%). Experimental results of 12-O-tetradecanoylphorbol-13 acetate (TPA) induced ear swelling validation mice model showed that ZOEOs treatment has better anti-inflammatory effect compared with ibuprofen (positive control) at high concentrations. Histological and immunohistochemical analysis showed that ZOEOs significantly decreased COX-2, IL-6 and NF-κB expression in a dose dependent manner. The mRNA levels of COX-2 and NF-κB were also down regulated by the application of ZOEOs. This indicated that ZOEOs exhibited positive effects in ear skin protection. Antibacterial experimental results showed that EOZOs had anti-bacterial effects on *Escherichia coli, Pseudomonas aeruginosa*, and *Staphylococcus aureus*. DPPH radical scavenging, A549 cell line and LNCaP cell line inhibition results indicated that ZOEOs exhibited potential antioxidant and anti-tumor properties. The findings of these study provide scientific basis on therapeutic use of ZO in food, cosmetic and pharmaceutical industries.

## Graphical Abstract

The essential oils of Z. officinale exhibited anti-inflammatory effects on TPA induced ear swelling in mice, antibacterial effects on different bacteria, anti-oxidant effect on DPPH free radical scavenging and anti-tumor property on human lung cancer A549 and human prostate cell lines LNCaP. 
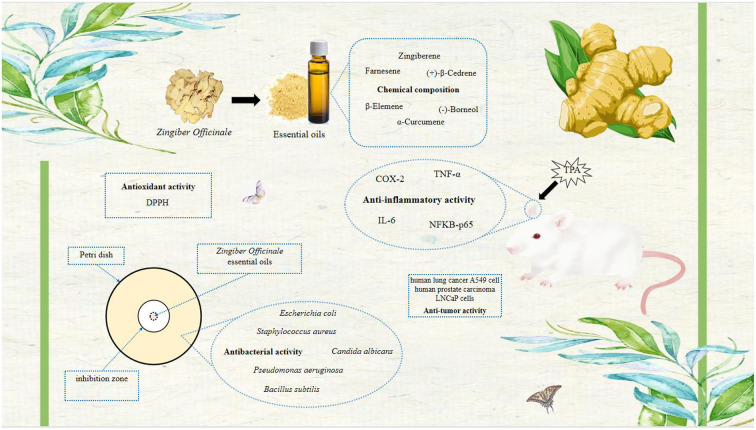

## Introduction

*Zingiber officinale* (ZO), has been used as a table condiment since ancient times and can be used for both meat and vegetables. ZO plays an important role in food safety, flavoring, and deodorization (1). In addition to that, ZO is reported to exhibit bioactive effects, such as antioxidation, weight loss, cold treatment, antiemetic effects, phlegm elimination, coughing relief and other health benefits properties (2–5). Additionally, ZO is mainly found in subtropical and tropical Asia, Africa, Far East Asia, China, and India (6, 7). The extracts of ZO have complex and diverse chemical compositions, among which, more than 400 compounds have been reported, mainly including carbohydrates, lipids, terpenoids and phenols (8). ZOEOs extract has large amounts of chemical compositions. A total of 43 ingredients have been previously reported in ZOEOs, and their skin protective effects were investigated (9). In addition to that, several previous studies have been carried out on the investigation of biological activities of ZOEOs (10–12). It was also found that ZOEOs exhibited DPPH radical scavenging capacities (13), anti-tumor effects (14), and antibacterial effects (15). However, ZOEOs derived from different extraction methods led to a variety of biological effects against varying pathologies (11, 16, 17). Inflammation is a driving factor of multiple diseases. ZO also confers a crucial inflammatory effect. It was reported that ginger essential oil extract could reduce the pro-inflammatory molecules to attenuate arthritis by lowering rheumatoid arthritis factor, C-reactive protein, and erythrocyte sedimentation rate level in the blood (18). Additionally, Cakir et al. (10) reported that ZOEOs showed positive effects on treatment and prevention of necrotizing enterocolitis *via* inhibiting inflammatory factors such as IL-6, P65 and COX-2. However, these activities are attributed to the active ingredients isolated from ZOEOs, which work synergistically to confer variety biological activities, and other special substances. Additionally, different extraction methods would lead to variety components of ZOEOs.

As we all known, ZO is widely used in food, cosmetics, and health products. However, the comprehensive investigation of bioactivity properties exhibited by ZOEOs are still unclear and the mechanisms of action remain unknown. This limits the utilization of ZOEOs. Therefore, in this study, the mechanism underlying the effects of essential oil of fresh ZO obtained by steam distillation on TPA induced ear inflammation mice were investigated. Although the photo aging skin protective effects of essential oil obtained from Ginger was illustrated, the effects of ZOEOs on ear skin inflammation was rarely reported. Furthermore, the antibacterial property was also determined by investigating the inhibitory effects of ZOEOs on five bacteria: *Staphylococcus aureus, Pseudomonas aeruginosa, Escherichia coli, Bacillus subtilis*, and *Candida albicans*. Additionally, antioxidant and anti-tumor capacities were also studied. Thus, we assumed that ZOEOs may represent a novel alternative agent for the alleviation of ear inflammation, and possesses multi-functionality applied in food, cosmetics and pharmaceuticals.

## Materials and methods

### Plant materials and chemicals

ZO, a plant material used in this research, was obtained from Guangzhou tongrentang pharmaceutical Co., Ltd (location 23°N and 118°E). ZO was processed in a series of ways, and then essential oil was extracted and used as the experimental material. 12-O-Tetradecanoylphorbol-13-Acetate (TPA) and DPPH were purchased from Shanghai Aladdin Biochemical Technology Co., Ltd. (Shanghai, China); 3,3'-Diaminobenzidine (DAB), Anti-TNF-α Rabbit pAb, Anti-IL-6 Rabbit pAb, Anti-NF-κB Rabbit pAb, Anti-COX-2 Rabbit pAb, HRP conjugated Goat Anti-Rabbit IgG (H + L) were purchased from Wuhan Servicebio Technology Co., Ltd. (Wuhan, China).

### Extraction of essential oil

ZO was already dried and sliced. The plant material was crushed and sieved using a sieve (with the aperture size of 0.45 mm). ZO was then extracted by steam distillation, and the whole extraction process took 3.5 h (19). After extraction, essential oil was separated from the oil-water mixture, dried with anhydrous sodium sulfate, and placed in a dark test tube to protect it from sunlight. The oil was stored was refrigerated at 4°C for further experiments (20, 21).

### GC-MS analysis

GC-MS analysis of ZOEOs was carried out using Focus GC model (Thermo Electron Corporation, USA). ADB-5 capillary column (Agilent, Santa Clara, CA, USA) of 30 m × 0.25 mm in size and 0.25 mm in thickness was used for analysis. Equipment operating conditions were as follows: oven temperature program: the initial temperature was set at 4°C, a constant temperature was maintained for 1 min, and gradually increased to 280°C at a rate of 5°C/min; injector and splitter temperature was: 220°C; with He used as the carrier gas. The split ratio was set as 1:10, and the flow rate was 1.0 mL/min. 1 μL sample was diluted in n-hexane with a volume ratio of 1:10. Results obtained were further analyzed to identify each compound. Identification methods were as follows: results were compared with a homologous series of n-alkanes (C_6_-C_40_); compared with data from literature and further compared with data presented in the National Institute of Standards and Technology (NIST) Chemistry Web Book (22, 23).

### TPA induced ear swelling validation mice model

#### Mice administration

Mice (6-8 weeks old, 18-20 g body weight) were purchased from Guangdong Experimental Animal Center. Animal studies were approved by animal experimental center of Sun yat-sen university. All procedures used in this study followed relevant ethical and institutional guidelines (SCXK/20130002, Guangzhou). Mice were grouped and raised in different cages and fed on a standard laboratory diet. The feeding period was at least 7 days. The feeding environment was a room with controlled conditions. Temperature was set at 23°C, air humidity was set at 60%, and the day-night cycle was 12 h. Mice were divided into 6 groups (*n* = 15): control group, model group (TPA treated group, 50 ng/mL), positive control group (TPA + ibuprofen 100 mg/kg), ZOEOs-L group (TPA + ZOEOs 25 mg/kg), ZOEOs-M group (TPA + ZOEOs 50 mg/kg) and ZOEOs-H (TPA + ZOEOs 100 mg/kg). Among them, TPA-acetone was applied to the ear surface of mice fully and evenly. After 30 min, different concentrations of essential oil solution were applied to the TPA-treated ear surface.

#### Determination of ear inflammatory level

After 6 h, mice were *via* cervical dislocation, and 6 mm diameter ear tissue was harvested, perforated, and weighed. The weights were recorded and the inhibition rate (%) was calculated according to the following formula (24).


$$\text{Inhibition }(\%)=[1-({\text{W}}_{\text{drug group}}{-\text{W}}_{\text{control}})/({\text{W}}_{\text{TPA group}}{-\text{W}}_{\text{control}})]\times 100$$

where *W*_drug group_ = weight of the ear with the sample or TPA treatment, *W*_control_ = weight of the ear in control group, *W*_TPA group_ = weight of the ear with the TPA treatment.

#### Histology and immunohistochemistry

Ear tissue of the mice was fixed with formaldehyde, then embedded in paraffin, and prepared into paraffin-embedded sections (4 μm). After dewaxing and rehydration, hematoxylin-eosin (HE) was used for staining. For immunohistochemical analysis, tissue sections were incubated overnight at 4°C with primary antibodies (1:200) diluted in PBS. Antibodies used in this experiment included cyclooxygenase 2 (COX-2), tumor necrosis factor (TNF-α), interleukin 6 (IL-6) and nuclear factor κB (NF-κB/P65). Tissues were sliced and incubated with biotin horseradish peroxidase antibodies (diluted 1:2,000) under 25°C for 1 h. After 1 h, sections were developed with 3,3′-diaminobenzidine. Immunolabeled sections were observed using a fluorescence microscope (Olympus, Japan) and the number of positive cells was determined using ImageJ™, NIH, Bethesda, MD, USA.

#### RNA isolation and gene quantification by RT-qPCR

RT-qPCR was applied to quantify the transcription gene levels of inflammatory cytokines (COX-2 and NF-κB/p65). Briefly, total RNA of tissues was extracted by using Trizol reagent according to manufacturer's protocol. The c DNA was synthesized by reverse transcription reaction, and then amplified by real-time fluorescence quantitative PCR and thermal cycler. Real-time PCR was measured by SYBR Premix Ex Taq on gene amplifiers in Hema Medical Instrument Co., Ltd (Zhuhai, Guangdong, China). The forward and reverse primers used were as follows: COX-2 (5′-TTCAACACACTCTATCACTGGC-3′ and 5′-AGAAGCGTTTGCGGTACTCAT-3′); NF-κB(p65) (5′-AGGCTTCTGGGCCTTATGTG-3′ and 5′-TGCTTCTCTCGCCAGGAATAC-3′); and GADPH (5′-AGGTCGGTGTGAACGGATTTG-3′ and 5′-TGTAGACCATGTAGTTGAGGTCA-3′). The mRNA expression was calculated as a fold change of gene expression.

### Antimicrobial assay

Antibacterial activity of ZOEOs was evaluated by disc diffusion method (25). Strains used to study antibacterial effect were: *S. aureus* (ATCC6538), *P. aeruginosa* (ATCC15442), *E. coli* (ATCC25922), *B. subtilis* (ATCC6633), and *C. albicans* (ATCC10231). The five strains were first cultured under the same conditions, then the same amount of essential oil was added to each, then cultured under appropriate conditions. After culturing, colonies obtained from the treatment were photographed, observed, and analyzed to assess antibacterial effect of ZOEOs.

#### Inhibition zone

Around 100 μL of each bacterial (108 CFU/mL) or fungal suspensions for fungi (104 spore/mL) was inoculated on the nutrient agar media (NA) or potato dextrose agar media (PDA). The sterile filter paper disc (6 mm diameter) was impregnated with 3 μL of each ZOEOs, and then aseptically placed on inoculated plates. After 24 (for bacteria) or 72 h (for fungi) of incubation at 37°C, the inhibition zones against tested strains were measured according to the inhibition halo formed around the disc (21).

#### Scanning electron microscopy (SEM) evaluation

The scanning electron microscopy (SEM) was performed as reported by Bismelah et al. (26) with some modifications. The bacteria suspensions were then centrifuged at 10,000×g for 10 min and the supernatant was removed to obtain the pellet. The bacteria pellet was then fixed with 2.5% glutaraldehyde in 0.1 M phosphate buffer pH 7.2 for a minimum of 2 h. The centrifugation was repeated three times. After centrifugation, the pellet was suspended in distilled water before undergoing the dehydration process for 10 min using two rounds of 30, 50, 70, 90, and 100% ethanol. The cells were then allowed to dry at 25°C before being mounted onto SEM stub and sputter-coated with gold. The samples were prepared according to the method and then examined under the SEM (Hitachi TM3000 Tabletop Scanning Electron Microscope).

### Antioxidant activity

Antioxidant activity of ZOEOs was determined by DPPH assay (27). Essential oils of different concentrations were mixed evenly with DPPH methanol solution and incubated for 0.5 h at room temperature. Absorbance of the mixture was then measured using a uv-300 spectrophotometer at a wavelength of 517 nm. Absorbance values obtained were analyzed using GraphPad Prism 7.0 software to identify essential oil concentration corresponding to the 50% free radical elimination rate (IC_50_). Free radical scavenging activity of ZOEOs was calculated as a percentage of radical inhibition using the following formula:


$$\begin{array}{l} \text{DPPH radical scavenging activity}\left(\%\right)=\\ \left[1-\left({\text{A}}_{\text{essential oil}}-{\text{A}}_{\text{blank}}\right)/{\text{A}}_{\text{control}}\right]\times 100 \end{array}$$

where *A*_essential oil_ = absorbance of the mixture of the essential oils sample and DPPH solution, *A*_blank_ = absorbance of the essential oils without the DPPH solution, *A*_control_ = absorbance of the DPPH solution.

### Effects of ZOEOs on A549 and LNCaP cells

#### Cell culture and treatment

The human lung cancer A549 cell line was purchased from the Type Culture Collection of the Chinese Academy of Sciences, Shanghai, China. A549 cells were cultured in DMEM supplemented with 10% fetal bovine serum (FBS) and penicillin (100 U/mL)/streptomycin (100 U/mL) at 37°C in a humidified atmosphere with 5% CO_2_. The human prostate cell lines LNCaP was obtained from the American Type Culture Collection. LNCaP cells were cultured in RPMI 1,640 supplemented with 10% fetal bovine serum (FBS), 100 mg/mL streptomycin, and 100 units/mL penicillin. Cells were maintained in a humidified incubator at 37°C with 5% CO_2_.

#### *In vitro* cytotoxic activity

The proliferation rates of lung cancer A549 cells and prostate cancer cells (LNCaP) in the presence of essential oils were determined by the colorimetric MTT assay according to previous study (28). Briefly, the cells (2.5 × 10^4^ cells/well) were seeded into 96-well microplates, and then essential oils with various concentrations (from 0 to 1 mg/mL) were added into the plates and incubated at 37°C for 24 h. After cells were incubated with MTT and maintained in a CO_2_ incubator for 3 h at 37°C in the dark, the absorbance was measured at 570 nm by a microplate reader. The inhibition ratio (*I* %) was evaluated using formula below,


$$\begin{array}{l} \text{I}\%\;=\;\left[\left(\text{A blank}-\text{A sample}\right)/\text{A blank}\right]\;\times 100\% \end{array}$$

where solution without essential oil was used as blank (Ablank) and the solution containing essential oils was used as the sample (Asample). The IC_50_ value of MTT assay was defined as the concentration of essential oils resulting in a 50% reduction of absorbance compared with blank.

### Statistical analysis

Data were obtained from at least three independent experiments. SPSS 19.0 (Chicago, USA) software was used to analyze data. ANOVA was used for continuous data to explore differences between groups. Standard error and significant differences were determined by Duncan's test. *P*-values < 0.05 or 0.01 were considered statistically significant.

## Results and discussion

### Chemical components of ZOEOs by GC-MS

Chemical constituents of ZOEOs were qualitatively analyzed by GC-MS, and 41 chemical constituents were obtained. Among the 41 components, sesquiterpenoids were the most abundant, accounting for 72.29% of the total content, with about 19 components. Monoterpenoids were the second abundant components, accounting for 14.93% of the total components, with 10 components. The yield of chemical composition analysis was 99.52%, and the main chemical components were: Zingiberene (19.71%), (+)-β-Cedrene (12.85%), Farnesene (12.17%), α-Curcumene (10.18%), β-Elemene (3.54%) and (–)-Borneol (2.73%). Some of the chemical components are shown in Table 1. Zingiberene, the most abundant compound among the six active ingredients, exerted effects against *in vitro* and *in vivo* human colon cancer cell growth *via* autophagy induction (46). Notably, (+)-β-Cedrene and α-Curcumene were reported to have significant antibacterial effect (47), which were considered as effective bacteriostatic components. Although the proportion of (–)-Borneol in ZOEOs was relatively low, studies report that this component has good bacteriostatic effect (48). The effects of these active ingredients show that ZOEOs has inhibitory effect against both bacteria and tumor, therefore further studies should be carried out to explore these effects.

**Table 1:** Chemical composition, retention index (RI) and relative content (%) of *Z. officinale* essential oil.

**No**.	**Compounds i**	**RI ii**	**Exp.RI**	**Ref**.	**Relative content (%)**
					***Z. officinale Roscoe***
1.	Cineole	1,059	1,044	a	1.06
2.	Linalool	1,082	1,082	a	1.43
3.	(–)-Borneol	1,138	1,160	b	2.73
4.	(–)-Terpinen-4-ol	1,137	1,160	c	0.37
5.	α-Terpineol	1,143	1,179	d	1.98
6.	Neral	1,174	1,207	a	2.40
7.	Citral	1,174	1,240	e	2.68
8.	L-Bornyl acetate	1,277	1,288	e	1.23
9.	2-Undecanone	1,251	1,291	f	1.04
10.	(+/–)-δ-Elemene	1,377	1,331	g	0.40
11.	DL-citronellol acetate	1,302	1,354	e	0.65
12.	(+)-Cyclosativene	1,125	1,369	h	0.75
13.	α-Copaene	1,221	1,372	i	0.87
14.	Acetic acid geranyl ester	1,352	1,360	j	1.73
15.	β-Elemene	1,398	1,403	g	3.54
16.	(–)-α-Cedrene	1,403	1,433	j	0.79
17.	(–)-(7S)-Germacrene D	1,515	1,485	i	0.37
18.	(+)-γ-Cadinene	1,435	1,497	j	0.25
19.	o-Menth-8-ene,4-isopropylidene-1-vinyl	1,431	1,434	k	1.94
20.	β-Sesquiphellandrene	1,446	1,524	g	1.51
21.	(+)-Aromadendrene	1,386	1,439	l	0.98
22.	(R)-β-Himachalene	1,528	1,499	m	1.66
23.	α-Curcumene	1,524	1,472	g	10.18
24.	Zingiberene	1,451	1,489	g	19.71
25.	Farnesene	1,458	1,497	g	12.17
26.	(+)-β-Cedrene	1,398	1,418	m	12.85
27.	(–)-Thujopsene	1,416	1,447	j	0.37
28.	Hedycaryol	1,694	1,530	e	1.15
29.	Nerolidol	1,564	1,552	b	2.46
30.	(+)-Viridiflorol	1,530	1,587	n	0.64
31.	(–)-Globulol	1,530	1,590	e	0.27
32.	α-Bisabolol	1,625	–	–	1.44
33.	γ-Eudesmol	1,626	1,593	e	0.32
34.	Guaiol	1,614	1,600	b	0.49
35.	12-Isopropyl-1,5,9-trimethyl-4,8,13-cyclotetradecatriene-1,3-diol	2,400	2,955	o	1.84
36.	β-Eudesmol	1,593	1,649	f	2.01
37.	4-Methyl-1-(6-methylhept-5-en-2-yl)cyclohex-3-en-1-ol	1,619	1,643	p	0.41
38.	α-Bisabolol	1,625	1,664	j	0.33
39.	2-Dehydrolinalool	1,090	1,068	j	1.13
40.	Geranyllinalool	2,046	2,444	e	1.14
41.	α-Bergamotene	1,430	1,434	q	0.25
	Total identified/%				99.52
	Total monoterpenoids/%				14.93
	Oxygenated monoterpenes/%				12.82
	Total sesquiterpenoids/%				72.29
	Oxygenated sesquiterpenes/%				8.61
	Others/%				12.3

^*i*^Compound listed in the order of elution from methyl silicone capillary column (30 m × 0.25 mm, 0.25 μm film thickness).

^*ii*^Retention indices (RIs) relative to n-alkanes (C_6_-C_40_) on the same methyl silicone capillary column.

^*a*^Hoskovec et al. (29); ^*b*^Ekundayo et al. (30); ^*c*^Orav et al. (31); ^*d*^Harangi (32); ^*e*^Munda et al. (33); ^*f*^Adams et al. (34); ^*g*^Angel et al. (35); ^*h*^Lucero et al. (36); ^*i*^Szafranek et al. (37); ^*j*^Tudor (38); ^*k*^Mohammed et al. (39); ^*l*^Rezazadeh et al. (40); ^*m*^Adams (41); ^*n*^Hazzit et al. (42); °Tao et al. (43); ^*p*^Senatore and Rigano (44); ^*q*^Viswanathan et al. (45).

### Effects of ZOEOs on ear weight of TPA induced mice ear

**Figure 1:**
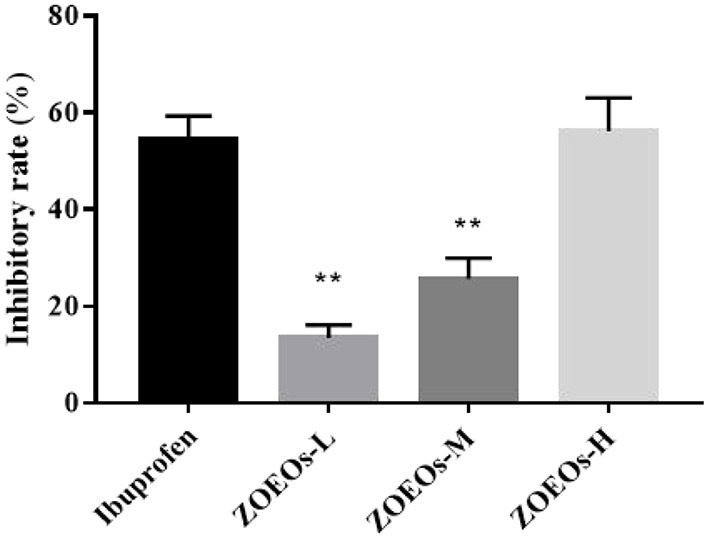
Inhibitory rate of mice ear weight in different groups. **ZOEOs vs. Model, *P* ≤ 0.01.

Treatment of mouse ear tissue with TPA for 6 h resulted in severe ear edema (Figure 1). As shown in Figure 1, ZOEOs showed good weight inhibitory rate. Especially, the high dose of ZOEOs (56.15%) reached a similar inhibitory rate of Ibuprofen (54.44%) at the same concentration. This indicated that ZOEOs treatment could reverse the ear inflammation induced by TPA.

### Effects of ZOEOs on histology of TPA-induced ear morphological changes

**Figure 2:**
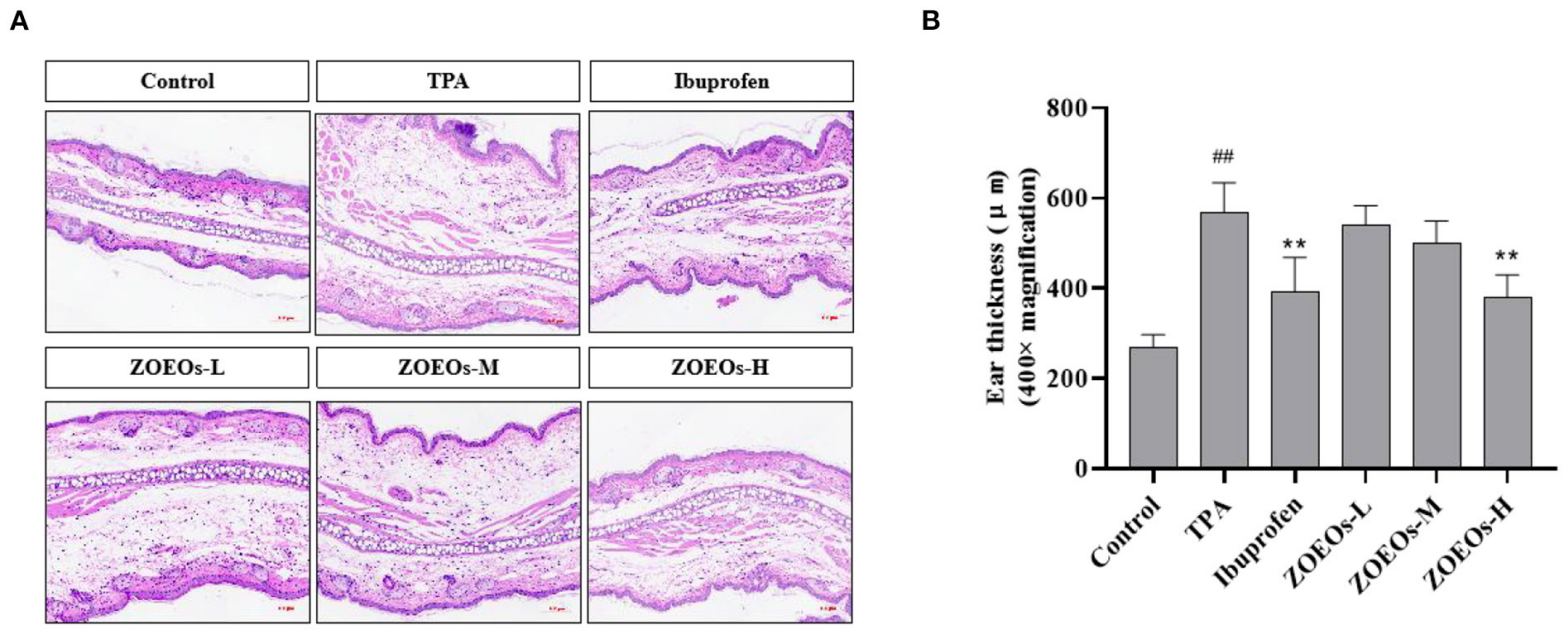
Histological sections of mice ear tissues in different mice groups **(A)** and the ear thickness **(B)**. ^##^Control vs. Model, *P* ≤ 0.01; **ZOEOs vs. Model, *P* ≤ 0.01.

According to the results of Figure 2, TPA treated mice showed a severe inflammation in ear which could be observed by HE staining (Figure 2A) and the ear thickness (Figure 2B; *P* < 0.01). Obviously, the treatment Ibuprofen significantly reduced the ear thickness, alleviating ear swelling response. A dose dependent manner and reduction of ear thickness were observed among the ZOEOs treatment groups. However, only ZOEOs-H exhibited remarkably inhibitory effects which was consistent with the preceding results (Figure 1). It could be seen that ZOEOs exhibited anti-inflammation effects in TPA induced ear inflammation.

### Effects of ZOEOs on immunohistochemistry of TPA-induced inflammation in ear

**Figure 3:**
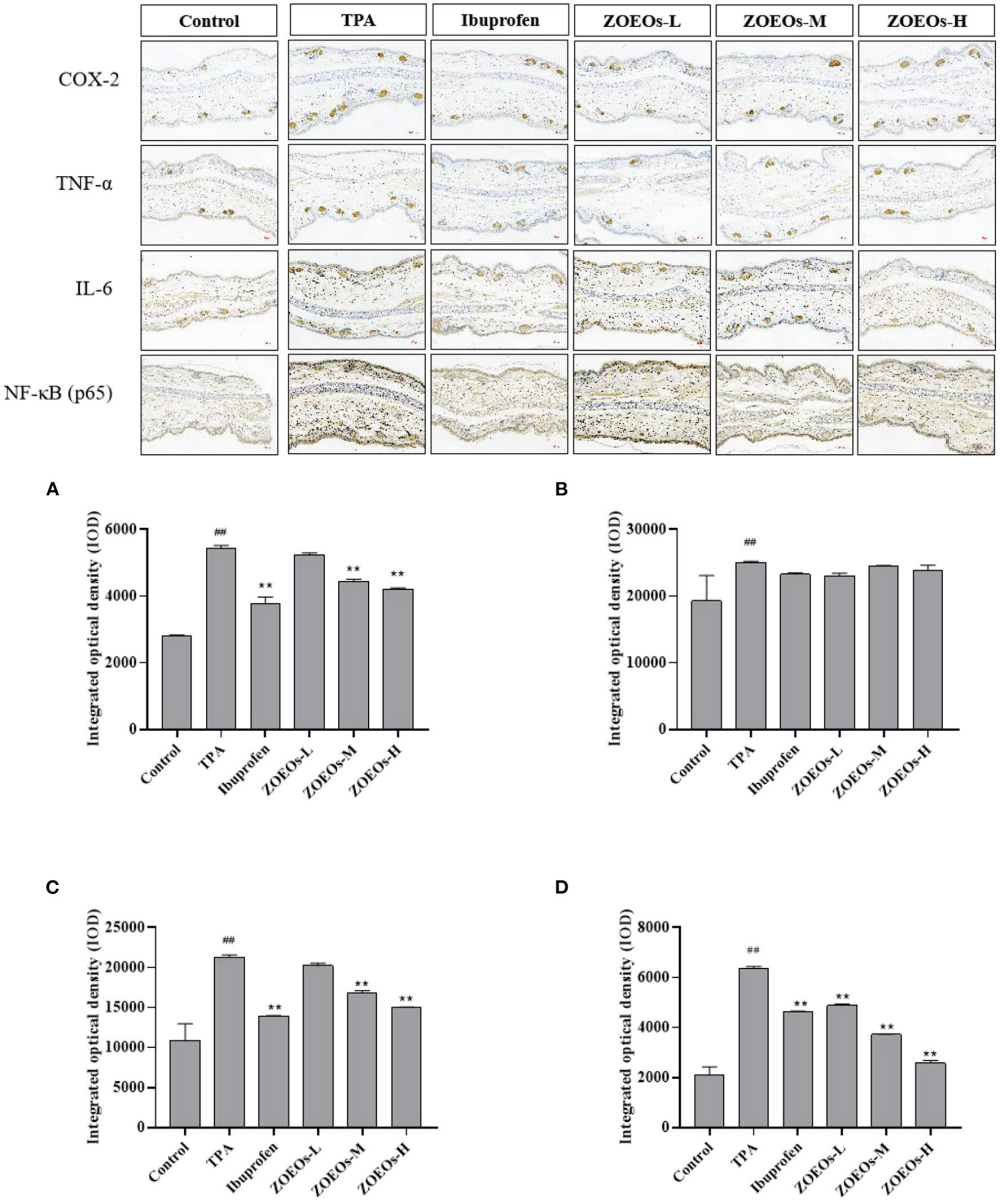
Immunohistochemical staining of mice ears of COX-2, TNF-α, IL-6 and NF-κB (P65) and the determination of levels for COX-2 **(A)**, TNF-α **(B)**, IL-6 **(C)** and NF-κB (P65) **(D)**. ^##^Control vs. Model, *P* ≤ 0.01; **ZOEOs vs. Model, *P* ≤ 0.01.

Inflammation was accompanied by production of inflammatory cytokines, such as TNF-α, IL-6, and COX-2, which can all be activated by TPA (49, 50). Expression of inflammatory cytokines is a biomarker indicating severity of inflammation. Therefore, immunohistochemical analysis was used to determine its expression levels of these cytokines. TPA treatment significantly increased expression levels of COX-2, TNF-α, IL-6, and NF-κB (P65) (*P* < 0.01), which could be reversed by the application of ibuprofen (Figure 3). Additionally, treatment with ZOEOs could significantly reduce the expression of NF-κB (P65) in a dose dependent manner (*P* < 0.01, Figure 3D). Especially, the alleviation effects of ZOEOs-M/H were better than that of ibuprofen. Whereas ZOEOs-M and ZOEOs-H application could remarkably inhibit the expression of COX-2 (Figure 3A) and IL-6 (Figure 3C; *P* < 0.01) except for ZOEOs-L. However, the significant alleviation effects of ZOEOs on TNF-α could not be observed.

Cytokines are involved in many biological processes including inflammation, including pro-inflammatory (TNF-α, IL-6, and IL-1β) and inflammatory cytokines (NF-kB, COX-2). Due to the key role in regulation and expression of pro-inflammatory cytokines, such as IL-6 and TNF-α, NF-κB is pivotal in initiating and amplifying inflammation response (51). According to the results, these findings implied that ZOEOs could attenuate TPA induced ear edema in mice *via* through reduction of TNF-α, IL-6, NF-κB, and COX-2. It was reported that eriocitrin and resveratrol could also relieve TPA-induced mouse ear edema through decreasing the levels of the pro-inflammatory cytokines TNF-α and IL-1β (52). Zhang et al. (53) investigated bioactivity of Curcuma phaeocaulis Valeton, indicating that essential oils derived from it showed markedly down-regulation effects of the expression of COX-2 and TNF-α. In addition, essential oil from ZO extracted with the same hydro-distillation method, was reported to be effective in ameliorating UVB-induced skin inflammation and inhibited IL-1β and TNF-α expression in skin tissues (9). These might all due to the anti-inflammation components of essential oils which might benefit its ear skin damage.

### Effects of ZOEOs on mRNA expression levels of inflammatory cytokines

**Figure 4:**
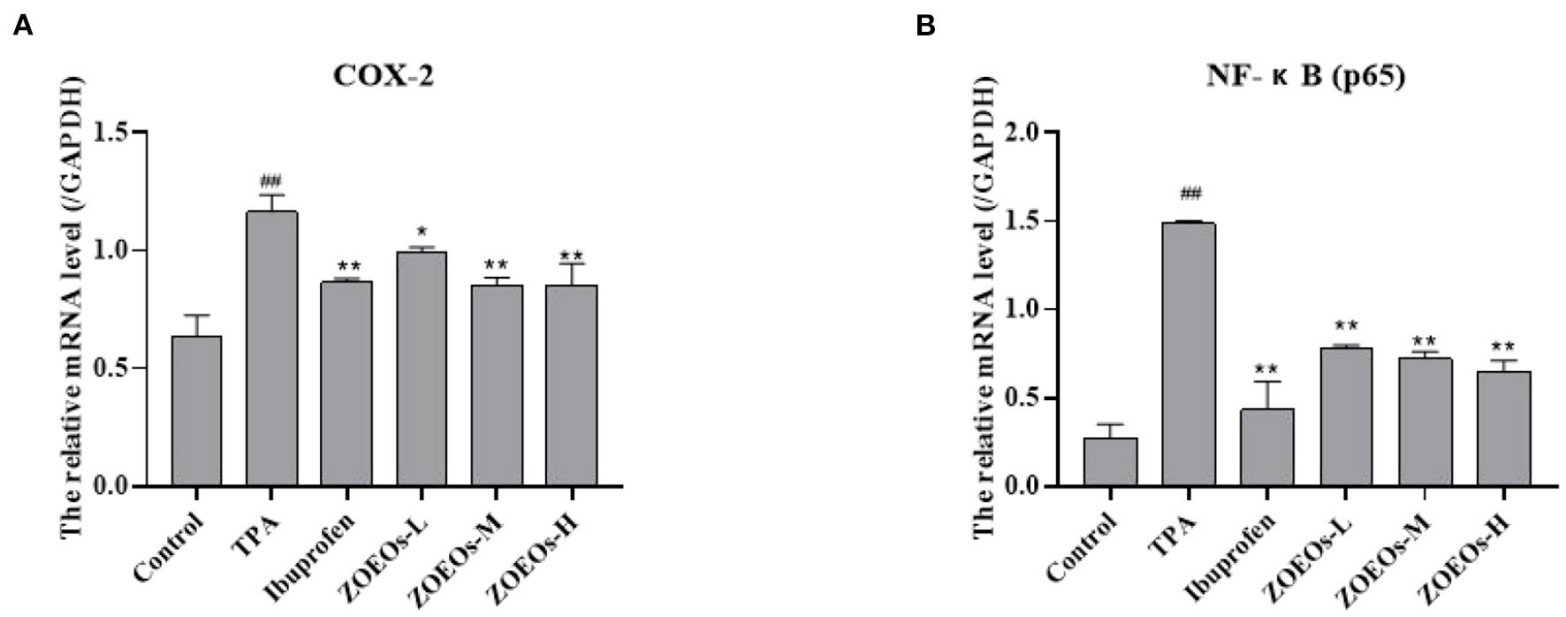
Effects of ZOEOs on mRNA level [**(A)**: COX-2, **(B)**: NF- NF-κB (P65)] of inflammatory factors of mice ear tissues for different groups. ^*##*^Control vs. Model, *P* ≤ 0.01; **ZOEOs vs. Model, *P* ≤ 0.01; *ZOEOs vs. Model, *P* ≤ 0.05.

In this study, real-time quantitative PCR was used to determine mRNA levels of various inflammatory factors (Figure 4). Inflammatory factors evaluated include COX-2 and NF-κB (p65). Results showed that TPA application could significantly upregulated the mRNA levels of COX-2 and NF-κB (p65). Both ibuprofen and ZOEOs treatment could significantly downregulated the expression levels. Intriguingly, the ZOEOs-M and H exhibit similar effects when compared with those of ibuprofen in the mRNA expression regulation of COX-2. Notably, reduction effect of essential oil treatment group on NF-κB (p65) was dose-dependent and inhibitory rate of both types of inflammations was more than 50%. Similarly, decursinol angelate was also reported to exert the same anti-inflammatory effects *via* regulating the expression of inflammatory cytokines (54). These findings show that ZOEOs have a good anti-inflammatory effect. Anti-inflammatory drugs have limitations such as gastrointestinal distress, kidney failure and heart failure. Therefore, several studies have been carried out to explore safe and effective anti-inflammatory drugs. ZOEOs provide a basis for the development of anti-inflammatory drugs for ear skin protection.

### Anti-bacterial effects ZOEOs

**Figure 5:**
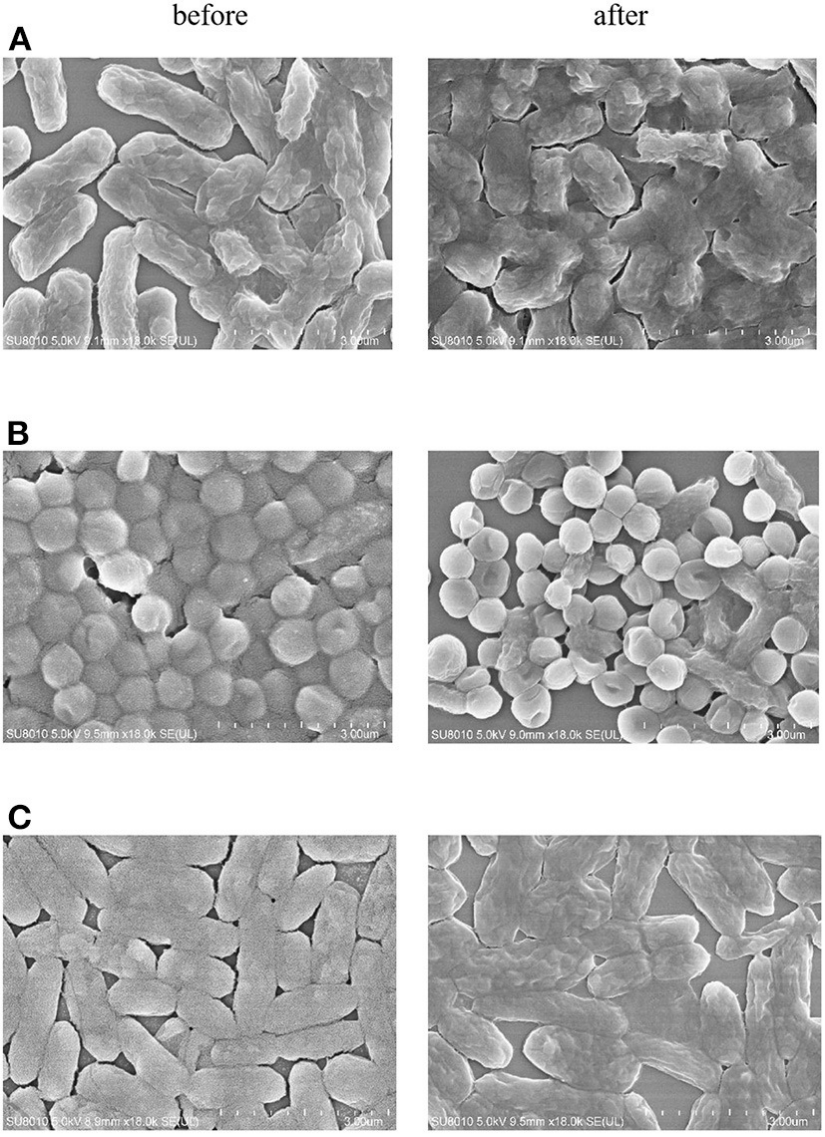
SEM micrograph of before and after treatment with ZOEOs in *E. coli* **(A)**, *P. aeruginosa* **(B)**, *S. aureus* **(C)** at scale bar of 3 mm. Under 18,000 × magnification, untreated bacterial cells remained intact and evenly distributed with no sign of morphological depression, whereas bacterial cells treated with ZOEOs depicted morphological disruption with blisters and deep craters on their surface.

The antimicrobial activities of ZOEOs were evaluated according to previous method (53). As shown in Table 2, the antibacterial effects of ZOEOs on gram-negative bacteria (*E. coli* and *P. aeruginosa*) and Gram-positive bacteria (*S. aureus*) was 0.56 ± 0.02, 0.42 ± 0.03, and 0.21 ± 0.02 mm, respectively. Unfortunately, no antimicrobial activities have been observed against *B. subtilis* (gram-posotive) and *C. albicans* (Fungus). In addition, it could be seen that the bacterial inhibitory effects of *Z. officinale* was not excellent according to the inhibition zone experiment. Beristain-Bauza et al. reported that monoterpenoids, sesquiterpenoids, phenolic compounds, and its derivatives, aldehydes, ketones, alcohols, esters et al. from ginger essential oils provided a broad antimicrobial spectrum against different microorganisms (11). This was in consistent with the GC-MS results of ZOEOs. The anti-bacterial effects of ZOEOs might be attributed to two main reasons: (a) the disruption of membrane and isolated mitochondria integrity and function; (b) the leakage of critical molecules and the inhibition of respiration and ion transport (11, 55, 56). This could be partially confirmed by the bacteria morphologic changes evaluated *via* scanning electron microscope (SEM). As shown in Figure 5, It was observed that for the bacteria (*E. coli, P. aeruginosa*, and *S. aureus*), each of them showed an intact cell wall and well-defined membrane and typical morphological appearance before exposure to ZOEOs. After the treatment of ZOEOs, some cells showed morphological destruction with blisters and craters on the surface of the cells. Some cells appeared as distressed revealing cell membrane indentations. Apparently, disruption of the membrane integrity was observed. It was reported that Cassia fistula Linn. stem bark extracts showed antibacterial effects on *E. coli* and *P. aeruginosa*. Lee et al. (1) investigated the antibacterial effect of extracts from different parts of ZO on *S. aureus* (*S. aureus*), showed that leaf, stem, and root extracts had 3, 3, and 2 mm clear zones, respectively. Similarly, our results illustrated that EOZOs had anti-bacterial effects on *E. coli, P. aeruginosa*, and *S. aureus*. This was also found in essential oils from *Curcuma. phaeocaulis* Valeton rhizomes (53).

**Table 2:** The diameter of inhibition zone of ZOEOs on different bacteria.

**Essential oils**	**Diameter of inhibition zone*****^*****a*****^*** **(mm)**				
	**Gram-negative**		**Gram-positive**		**Fungus**
	***E. coli***	***P. aeruginosa***	***S. aureus***	***B. subtilis***	***C. albicans***
ZOEOs	0.56 ± 0.02*^*c*^*	0.42 ± 0.03	0.21 ± 0.02	–*^*b*^*	–*^*b*^*

^*a*^The diameters of the inhibition zones excludes the diameters of disks (6 mm).

^*b*^No efficient antimicrobial activities against tested bacteria or fungus.

^*c*^Date are means ± SD of three replications.

### Antioxidant activity of ZOEOs

**Figure 6:**
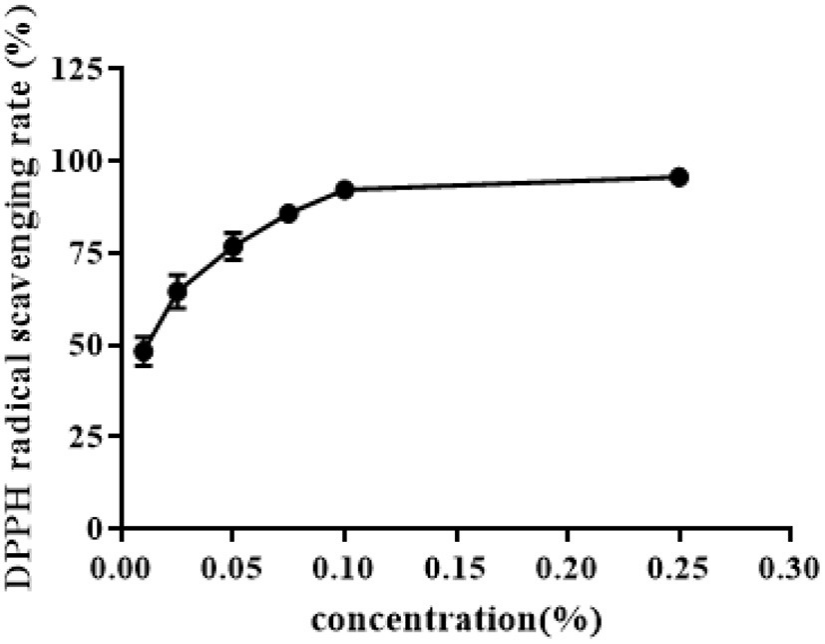
DPPH radical scavenging capacity of ZOEOs.

The scavenging rate of ZOEOs against free radicals was determined by DPPH method to show the antioxidant activity of ZOEOs. Data obtained from the experiments were analyzed and were presented in Figure 6.

DPPH radical clearance rate of ZOEOs increased with increase in concentration, DPPH clearance rates of ZOEOs within the experimental concentration range, were all higher than 45%. Data analysis showed that free radical scavenging rate reached 50% when the concentration of ZOEOs was at 0.01%. With the increase of concentration, the scavenging rate increased significantly, up until 0.1%. During the range between 0.1 and 0.25%, the rate reached a plateau. At the concentration of 0.25%, it showed the highest free radical clearance rate at ~95%, which almost achieved complete free radical scavenging effect. ZOEOs at a concentration of 0.01% showed the lowest scavenging rate, at about 46%. The difference between the highest and lowest scavenging effect was about 49%. These results indicated that ZOEOs exhibited excellent antioxidant capacity. This was in line with results studied by Li, who made comparisons of antioxidant activities among fresh, dried, stir-frying, and carbonized ginger. The highest DPPH radical scavenging rate was 90.12% which was observed in dried ginger (6), and lower than that in our study. Additionally, Camargo reported that different extraction methods, like solvent (?methanol and ethanol), ratio (70:30 and 95:5) and temperature (40 and 80°C) led to varied DPPH radical scavenging rate. The variation range was 40–96%. The different results were resulted from different extraction method (hydrodistillation was applied in our study), which would result in different components (12). Therefore, the bioactivity changes as the components vary. The specific effects of ZOEOs in oxidative stress of certain health-related model should be further investigated.

### Anti-tumor effects of ZOEOs on A549 and LNCaP cells

**Figure 7:**
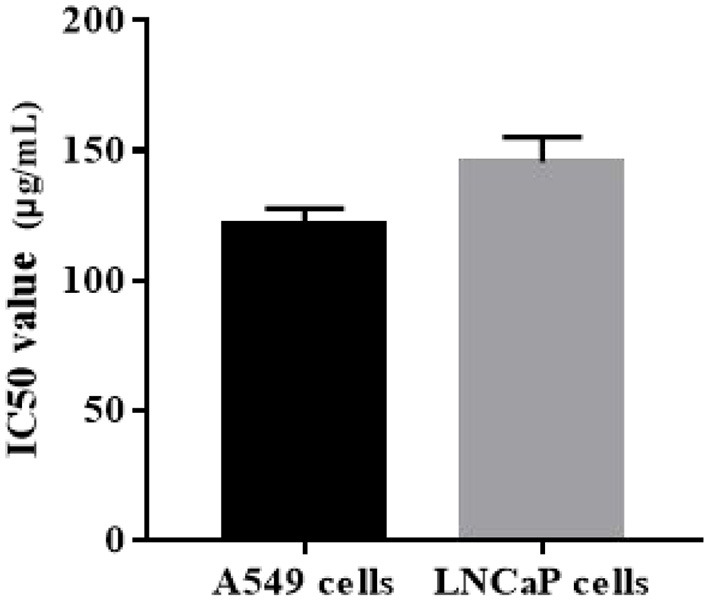
Anti-tumor properties (human lung cancer A549 and human prostate cell lines LNCaP) of ZOEOs.

Cytotoxicity effects of ZOEOs were evaluated using human lung cancer A549 cells and human prostate carcinoma LNCaP cells *in vitro*. Evaluation of cytotoxicity of ZOEOs was used to reflect its antitumor activity. Anti-tumor activity was demonstrated by half inhibition rate of essential oil on cell growth (IC_50_), and the effects are shown in Figure 7. the IC_50_ value was 121.52 ± 6.16 and 145.50 ± 9.65 μg/mL, respectively.

Ginger rhizome is widely cultivated both as spice and traditional cure for certain diseases (8). It noteworthy that ginger also exhibits anticancer properties in variety experimental models, including colorectal cancer HCT-116 cell line (57), diethylnitrosamine (DEN)-induced liver cancer (58), human cervical cancer HeLa cells and others (8). In this study, we determined the anti-tumor effects of ZOEOs on A549 and LNCaP cells. Czerwonka et al. (59) investigated the anti-cancer effects of the water extract of a commercial Spirulina (*Arthrospira platensis*) product on the human lung cancer A549 cell line, showing that The IC_50_ value was estimated at 99.2 μg/mL. Similarly, three Thai herbs, *Bridelia ovata, Croton oblongifolius*, and *Erythrophleum succirubrum* were extracted by ethyl acetate and 50% ethanol, and the IC50 values for A549 were all above 200 μg/mL, except for *C. oblongifolius* (118.41 ± 8.07 μg/mL) (60). The IC50 value of ZOEOs for A549 was in between, indicating a good anti- human lung cancer function. Additionally, as a human prostate carcinoma, LNCaP was widely used and investigated. Kazemi et al. (61) reported that the IC50 value of flavonoids in *Alpinia officinarum Hance*. was 168 μg/mL, which was higher than that of ZOEOs. These all showed that ZOEOs exhibited good anti-cancer properties, which might be related to its special components, such as zingiberene.

## Conclusion

The chemical composition, anti-inflammation, antibiosis, antioxidation, and antitumor activity of ZOEOs were evaluated. Essential oil was extracted from ginger by steam distillation, and a total of 41 compounds of the four essential oils were analyzed using GC-MS. The main chemical components identified included Zingiberene (19.71%), (+)-β-Cedrene (12.85%), Farnesene (12.17%), α-Curcumene (10.18%), β-Elemene (3.54%) and (–)-Borneol (2.73%).

Anti-inflammatory effects of ZOEOs were assessed using a TPA induced ear swelling validation model. The results from anti-inflammatory experiments and GC-MS analysis showed that ZOEOs have active compounds that significantly improve auricle swelling and reduce expression of inflammatory factors. Analysis showed that ZOEOs have significantly higher anti-inflammatory activity compared with ibuprofen. Furthermore, analysis shows that ginger essential oil has a high inhibitory effect on certain bacteria. In addition, antioxidant activity of essential oils was evaluated through DPPH *in vitro* experiment. Analysis showed that ZOEOs exhibit excellent antioxidant effects at low concentrations. In addition, ginger essential oil showed anti-tumor activity.

In summary, ginger essential oil has a variety of activities. Thus, it has potential application in food, skin care cosmetics and health care products. Further, studies should be carried out to explore activities of active ingredients of ginger.

## Data availability statement

The original contributions presented in the study are included in the article/supplementary material, further inquiries can be directed to the corresponding author/s.

## Ethics statement

The animal study was reviewed and approved by Animal experimental Center of Sun Yat-sen University.

## Author contributions

SZ, TZ, LaZ, LiZ, and MY performed the experiments. SZ, TZ, LaZ, LiZ, MY, DL, and YZ analyzed the data and wrote the manuscript. TZ, LiZ, MY, SC, and WL analyzed and discussed the data. SZ, LaZ, and TZ provided samples. LaZ and TZ designed the research content and modified the manuscript. All authors have read and approved the final manuscript.

## Funding

This work was financially supported by grants from the National Natural Science Foundation of China (No. 32201980), Guangzhou Basic and Applied Basic Research Project (No. 202201010680), Chaozhou Branch of Chemistry and Chemical Engineering Guangdong Laboratory Funding (No. HJL202202B008), the Excellent Doctoral Talents Introduction Project of Guangdong Academy of Agricultural Sciences (R2021YJ-YB3006), the Guangdong Basic and Applied Basic Research Foundation (2020A1515110715), the Guangdong Provincial Key Laboratory of Plant Resources Biorefinery (2021GDKLPRB02), and the Guangdong Technology R&D Program (2021B0202060001).

## Conflict of interest

Author SZ was employed by Shenzhen Precision Health Food Technology Co., Ltd. The remaining authors declare that the research was conducted in the absence of any commercial or financial relationships that could be construed as a potential conflict of interest.

## Publisher's note

All claims expressed in this article are solely those of the authors and do not necessarily represent those of their affiliated organizations, or those of the publisher, the editors and the reviewers. Any product that may be evaluated in this article, or claim that may be made by its manufacturer, is not guaranteed or endorsed by the publisher.
